# Household food insecurity, dietary intake, and weight status among children in Madrid, Spain: A cross-sectional study

**DOI:** 10.1371/journal.pone.0358967

**Published:** 2026-09-30

**Authors:** Luis Carmona-Rosado, Julia Díez, Pedro Gullón, Elisa Buenaga, Ana Moragues-Faus, José Manuel Díaz-Olalla, Luisa N. Borrell, Manuel Franco

**Affiliations:** 1 Public Health and Epidemiology Research Group, School of Medicine and Health Sciences, Universidad de Alcalá, Alcalá de Henares, Madrid, Spain; 2 Graduate School of Public Health & Health Policy, The City University of New York, New York, United States of America; 3 Conselleria de Sanidad Universal y Salud Pública, Generalitat Valenciana, Valencia, Spain; 4 Departamento de historia económica, Instituciones, política y economía municipal, Universitat de Barcelona, Barcelona, Spain; 5 Madrid Salud, Ayuntamiento de Madrid, Madrid, Spain; 6 BC3 Basque Centre for Climate Change, Adaptation Lab, Leioa, Basque Country, Spain; 7 Department of Epidemiology, Johns Hopkins Bloomberg School of Public Health, Baltimore, United States of America; Federal University of Minas Gerais: Universidade Federal de Minas Gerais, BRAZIL

## Abstract

**Background:**

Household food insecurity (HFI) is increasingly prevalent and disproportionately affects households with children, who may be particularly vulnerable to adverse diet-related outcomes. However, evidence on the association between HFI and these outcomes in Mediterranean settings is scarce. This study assessed the association between HFI, dietary intake, and weight status in a socioeconomically diverse sample of children aged 3–12 years living in Madrid, Spain.

**Methods:**

We analyzed data from a citywide cross-sectional survey conducted in Madrid in 2017 (n = 5,144), including objective anthropometric measurements (height and weight) of children aged 3–12 years, and parent-reported information on HFI and children’s intake of fruits, vegetables, and sugar-sweetened beverages (SSBs). We fitted Poisson regression models with robust standard errors to estimate prevalence ratios (PRs) for overweight and obesity, and multinomial logistic regression models to estimate odds ratios (ORs) for dietary intake outcomes, with corresponding 95% confidence intervals (CIs).

**Results:**

Overall, 18.1% of children experienced HFI. After adjustment for age, sex, and family affluence, HFI was associated with a higher prevalence of overweight (PR  =  1.17; 95% CI: 1.04, 1.31), but not obesity (PR  =  1.20; 95% CI: 0.97, 1.48). Experiencing HFI was also associated with higher odds of never consuming fruits (OR= 1.81; 95% CI:1.25, 2.64) or vegetables (OR= 3.23; 95% CI: 2.03, 5.13), and of consuming SSB daily (OR = 1.67; 95% CI: 1.01,2.76). Moreover, we found stronger associations among girls; however, we only observed evidence of interaction by sex for fruit consumption.

**Conclusions:**

HFI was associated with a less frequent consumption of fruits and vegetables, a more frequent intake of SSBs, and a higher prevalence of overweight. Our findings underscore the need for policies and interventions addressing HFI and supporting access to healthy foods, particularly among socioeconomically disadvantaged households with children.

## Introduction

Food insecurity has been defined by the U.S. Department of Agriculture (USDA) as the ‘limited or uncertain availability of nutritionally adequate and safe foods or limited or uncertain ability to acquire acceptable foods in socially acceptable ways’ [1]. Notably, food insecurity is a major social, economic, and public health concern, including in high-income countries [2–5].

In the U.S., 18.4% of households with children experienced food insecurity in 2024 [6]. Similarly, 19.6% of Canadian households with children experienced food insecurity in 2022 [7]. Most studies on household food insecurity (HFI) have been conducted in North America, where nationally representative surveys have provided extensive evidence of its prevalence, determinants, and health consequences [8–11].

In Europe, research on the prevalence and distribution of HFI remains comparatively limited [12,13]. Previous studies, despite being heterogeneous, have indicated that southern European countries, including Italy, Portugal, and Greece, are among those most affected [14–17], whereas HFI appears to be less prevalent in Nordic countries [18]. In Spain, the only existing national study, conducted in 2021, estimated that 13.3% of households experienced HFI [19].

HFI is associated with social exclusion and material deprivation and may adversely affect children’s cognitive, emotional, and physical health and well-being [3,20]. HFI may also contribute to childhood overweight and obesity [21,22], although the evidence is inconsistent. Two recent systematic reviews found that some studies reported a higher prevalence of overweight and obesity among children experiencing HFI, whereas others reported inverse or null associations [23,24]. More recently, longitudinal evidence has documented that trajectories of food insecurity during childhood were associated with weight status and poorer self-reported health in young adulthood [25].

Most studies examining the association between HFI and childhood weight status have been conducted in North America, while European evidence is scarce [26]. Therefore, the extent to which HFI is associated with childhood overweight and obesity in Europe remains unclear. This is a particularly relevant concern in Spain, where most recent data show that approximately one in three children and adolescents have overweight, while one in ten lives with obesity [27].

Dietary intake may be one of the mechanisms underlying the association between HFI and childhood obesity [10,28]. For instance, food-insecure households may adopt purchasing strategies that prioritise affordability and meeting energy needs over nutritional quality, potentially increasing their reliance on energy-dense, nutrient-poor foods [29,30]. In Mexico, several studies have found associations between HFI and poorer dietary intake among children, including lower fruit and vegetable consumption and higher intake of energy-dense foods and added sugars [31,32]. In Greece, children aged 5–11 experiencing HFI had poorer dietary patterns than children living in food-secure households [33]. Similarly, Wu et al. found that dietary behaviours partly mediated the association between HFI and obesity among socioeconomically disadvantaged young people in Taiwan. Moreover, the associations of HFI with health outcomes may differ by sex [34]. While the evidence is more inconsistent for boys, some studies suggest that girls experiencing HFI may be more likely to develop overweight or obesity [35–37]. Potential explanations include differences in intrahousehold food allocation, responses to food scarcity, and eating behaviours, although the mechanisms remain insufficiently understood [38,39]. Evidence for these sex-specific associations in European populations is particularly limited. Given this evidence, we hypothesized that HFI would be more prevalent among children from less affluent households and would be associated with a higher prevalence of overweight and obesity, more frequent consumption of SSB, and lower intake of fruits and vegetables (F&V). We also hypothesized that these associations would be stronger among girls than among boys. Therefore, this study aimed to assess the associations of HFI with childhood obesity and dietary intake in a socioeconomically diverse sample of children aged 3–12 years living in Madrid, Spain, and to assess whether these associations differed by sex.

## Methodology

### Participants

We conducted a cross-sectional study following the Strengthening the Reporting of Observational Studies in Epidemiology (STROBE) Statement (see S1 File) [40]. We used de-identified data from the *“Encuesta sobre la situación nutricional de la población infantil en la Ciudad de Madrid”,* a city-wide representative survey of school-aged children aged 3–12 years living in Madrid [41]. This survey was conducted by the Madrid City Council in 2017 and collected detailed information on children’s sociodemographic characteristics, dietary intake, and anthropometric measurements.

Based on the initial sample size calculation, the target was to recruit 75 schools, approximately 375 classrooms, and a sample of 5,500 children. Children aged 3–12 years were recruited using a multistage cluster sampling design. First, schools were stratified by the socioeconomic characteristics of their surrounding areas. Of the 84 randomly selected schools invited, 60 agreed to participate (response rate: 71.4%). Within these participating schools, classes were randomly selected, and all children in the selected classes (n = 336) were invited to participate (n = 7,740). After excluding 196 children residing outside the municipality of Madrid, the final analytical sample comprised 5,961 children. Further details on the sampling strategy are available elsewhere [41].

For the present analysis, we included children for whom household food security status was available (n = 5,144). Subsequently, we excluded 308 children with missing information on family affluence. As the availability of outcome data differed across variables, our final analytical sample comprised 4,241 children for overweight and obesity, 4,769 for fruit intake, 4,763 for vegetable intake, and 4,770 for consumption of sugar-sweetened beverages (SSBs). S1 Fig, in the supplementary materials, provides the sampling selection process.

We conducted this study in accordance with the Declaration of Helsinki. Parents or legal guardians provided written informed consent prior to data collection. The Ethics Committee of the Universidad de Alcalá approved the secondary analysis of these data on October 28, 2019 (CEI/HU/2019/35). The authors had access to the data from July 16, 2020. This database was de-identified before being made available to the researchers, and the authors had no access to information that could identify individual participants.

## Measures

### Household food insecurity

Our exposure variable was household food insecurity (HFI), measured using the Household Food Insecurity Access Scale (HFIAS), a validated instrument designed to assess household experiences of food insecurity over the preceding four weeks [42]. The HFIAS was developed by the US Agency for International Development’s Food and Nutrition Technical Assistance Project and has been used to distinguish between food-secure and food-insecure households across different settings.

The HFIAS includes nine “occurrence” and nine corresponding “frequency-of-occurrence” questions covering three domains: anxiety or uncertainty about the household food supply, insufficient food quality, and insufficient food quantity during the preceding four weeks [42]. We classified the responses according to the standard HFIAS algorithm into four categories: food secure, mildly food insecure, moderately food insecure, and severely food insecure [42] (see S2 Fig). We also created a dichotomous HFI variable to classify households as either food secure or food insecure, with the latter category including mild, moderate, and severe HFI.

### Weight status

Our first set of outcome variables was overweight and obesity, derived from objectively measured height (cm) and weight (kg). Trained staff measured each child using standardised procedures and regularly calibrated equipment. Height was measured without shoes using a MZ 10042 stadiometer, and weight was measured in light clothing and without shoes using a TANITA UM-076 digital scale [41].

We calculated body mass index (BMI) as weight in kilograms divided by height in meters squared and derived age- and sex- standardised BMI z-scores using the World Health Organization (WHO) growth reference curves to derive weight status [43]. Consistent with these curves, we classified children as being overweight, including obesity, if their BMI z-score was greater than +1 standard deviation (SD) above the WHO reference median, and as having obesity if their BMI z-score was greater than +2 SDs above the WHO reference median. We analysed each outcome as a separate binary variable.

### Dietary intake

Our second set of outcome variables was the frequency of fresh fruit and vegetable consumption. We assessed these outcomes using responses from parents or legal guardians to two questions: *‘how many times a week does your son/daughter usually eat fresh fruits?*’ and *‘how many times a week does your son/daughter usually eat vegetables?*’ Response categories were never, 1–3 times/week, 4–6 times/week, and 7 times/week. We used “7 times/week” as the reference category because it represented the daily consumption that was most consistent with international and Spanish dietary recommendations [44].

The third outcome was the frequency of SSBs consumption. We measured it using parents’ responses to three questions regarding their child’s consumption of soft drinks, fruit drinks, and sugar-sweetened yogurt drinks. Based on the WHO definition, we combined these three beverage groups to derive an overall measure of SSB consumption by assigning each child the highest reported consumption frequency across beverage categories [45]. Response categories were never, 1–3 times/week, 4–6 times/week, and 7 times/week. We used “never” as the reference category, consistent with the recommendations to minimize the consumption of added and free sugars [44,46].

### Covariates

We included age (in years, based on the birth date reported in the survey), sex (boys or girls, because gender identity was not collected in the original survey), and family affluence as covariates [47,48]. Family affluence was measured using the Family Affluence Scale III (FAS-III) [49–51]. The FAS-III has demonstrated good reliability, construct validity, and cross-national comparability across diverse cultural and socioeconomic settings in Europe and North America [52]. This instrument consists of six indicators of material assets and living conditions: family car ownership, having one’s own bedroom, number of family holidays, number of bathrooms, ownership of a dishwasher, and number of computers. The FAS III score was calculated as the sum of the responses to these indicators, ranging from 0 to 13.

Following previous research and international guidelines [52–54], we used these scores to classify children in the lowest 20% (low family affluence, scores 0–6), the middle 60% (medium family affluence scores 7–9), and the highest 20% (high family affluence, scores 10–13). All covariates were self-reported by the children’s parents or legal guardians.

### Statistical analyses

We conducted descriptive statistics to characterise the study population, overall and by HFI status. We reported means and proportions with their corresponding 95% confidence intervals (CI). We used Poisson regression models with robust standard errors to estimate the associations of HFI with overweight and obesity. Because overweight and obesity were common in the study population, we reported prevalence ratios (PRs) and 95% CI. We used multinomial logistic regression models to examine associations of HFI with dietary intake consumption of fruits, vegetables, and SSBs. We reported odds ratios (OR) and 95% CI for each dietary-frequency category.

For each outcome, we first fitted an unadjusted model, followed by an adjusted model for age and sex. In addition, we fitted another model additionally adjusting for family affluence. We tested an interaction term between HFI and sex to assess whether the associations differed by sex. We then stratified and showed the results according to sex; these models were adjusted for age and family affluence only.

For dietary outcomes, we used the dichotomous HFI variable because the limited number of observations in some combinations of HFI severity and dietary frequency categories resulted in imprecise estimates. However, in the supplementary materials, we present the results of the associations using HFI with four categories.

We conducted complete case analyses for each outcome. We incorporated sampling weights in the analysis to account for the stratified cluster sampling design. We performed all analyses using R-4.1.2 software and its *survey* package [55].

## Results

### Descriptive statistics

Table 1 and S1 Table show the characteristics of the study population, overall and by HFI status. Overall, children had a mean age of 7.28 years, almost half were girls (48.6%), and the largest proportion lived in households of low family affluence (40.7%),

**Table 1:** Characteristics of participating children, stratified by household food insecurity, Madrid, Spain, 2017.

	Overall		Without HFI		Mild HFI		Moderate HFI		Severe HFI	
N (%)	5,144 (100.0)		4,168 (81.9)		305 (5.1)		347 (6.5)		324 (6.4)	
**Age, years (mean, SD)**	7.28 (2.84)		7.18 (2.82)		7.60 (2.69)		7.36 (2.99)		8.13 (2.86)	
**Sex, % (95% CI)**										
Girls	48.6	(47.1, 50.0)	48.5	(46.9, 50.1)	46.2	(39.9, 52.7)	46.0	(40.4, 51.8)	54.1	(48.4, 59.8)
**Family Affluence, % (95% CI)**										
Low	40.7	(39.2,42.1)	30.5	(29, 32.1)	81.4	(75.4, 86)	90.1	(86.1, 93.1)	92.3	(88.4, 95)
Medium	34.1	(32.6, 35.5)	39.4	(37.8, 41)	14.8	(10.6, 20.2)	7.7	(5.1, 11.4)	5.8	(3.5, 9.4)
High	25.3	(24, 26.6)	30.1	(28.6, 31.7)	3.8	(2.0, 7.4)	2.2	(1.0, 4.6)	1.9	(0.8, 4.4)
**Body weight, % (95% CI)**										
Overweight	25.4	(24.2, 26.7)	24.4	(23.1, 25.8)	35.4	(29.5, 41.8)	29.2	(24.3, 34.7)	26.2	(21.5, 31.6)
Obesity	15.4	(14.4, 16.5)	13.1	(12.1, 14.3)	23.7	(18.7, 29.6)	26.2	(21.5, 31.5)	26.6	(21.8, 32.0)
**Dietary intake, % (95% CI)**										
Daily consumption of fruits	32.6	(31.2, 34)	35.4	(33.9, 37.0)	18.2	(13.7, 23.8)	20.6	(16.4, 25.6)	19.4	(15.2, 24.4)
Daily consumption of vegetables	8.2	(7.4, 9.0)	9.1	(8.2, 10.1)	3.9	(2.1, 7.3)	2.9	(1.5, 5.5)	4.8	(2.8, 7.9)
No consumption of SSBs	6.3	(5.6, 7.1)	7.1	(6.3, 8.0)	2.8	(1.3, 5.8)	2.3	(1.1, 4.8)	3	(1.6, 5.7)

HFI: Household Food Insecurity; SD: Standard Deviation; CI: Confidence Interval; SSBs: Sugar-Sweetened Beverages

We found that 18.1% (95% CI: 17.0, 19.2) of children experienced HFI, with 5.1% living in mildly food-insecure households (95% CI: 4.4, 5.8), 6.5% in moderately food-insecure households (95% CI: 5.6, 7.7), and 6.4% in severely food-insecure households (95% CI: 5.4, 7.7). Among children living in food-insecure households, most lived in households with low family affluence (88.4%).

The prevalence estimates of overweight and obesity were 25.4% and 15.4%, respectively. The prevalence of obesity was 13.1% among children living in households without HFI, 23.7% among those in mild HFI, 26.2% among those with moderate HFI, and 26.6% among those with severe HFI. Moreover, a lower proportion of daily fruit and vegetable consumption was observed among children from food-insecure households compared to those without HFI.

### Household food insecurity and weight status

Table 2 shows the unadjusted and adjusted associations of HFI with overweight and obesity. Overall, we found a higher prevalence of overweight and obesity among children living in households with HFI. However, after adjusting for sex, age, and family affluence, the association remained statistically significant only for overweight among children with mild HFI (PR = 1.27, 95% CI: 1.04, 1.53). When we evaluated the associations using dichotomised HFI (data shown in S2 Table), we found that children living in food-insecure households had a statistically significant higher prevalence of both overweight (PR  =  1.50, 95% CI: 1.35, 1.66) and obesity (PR  =  1.97, 95% CI: 1.63, 2.39). After adjusting for sex, age, and family affluence, this remained statistically significant only for overweight (PR  =  1.17, 95% CI: 1.04, 1.31).

**Table 2:** Unadjusted and adjusted prevalence ratios (PRs) and their 95% confidence intervals (CIs) for the associations of household food insecurity (HFI) with overweight and obesity in children, Madrid, 2017.

	Overweight					
	Overall (n = 4,241)		Boys (n = 2,120)		Girls (n = 2,121)	
	PR^1^ (95% CI)^2^		PR^1^ (95% CI)^2^		PR^1^ (95% CI)^2^	
	Unadjusted	Adjusted^3,4^	Unadjusted	Adjusted^3,4^	Unadjusted	Adjusted^3,4^
Without HFI	1.00	1.00	1.00	1.00	1.00	1.00
Mild HFI	1.57 (1.31, 1.87)	1.27 (1.04, 1.53)	1.52 (1.19, 1.91)	1.12 (0.86, 1.44)	1.63 (1.24, 2.11)	1.45 (1.08, 1.90)
Moderate HFI	1.48 (1.25, 1.73)	1.14 (0.95, 1.36)	1.50 (1.20, 1.84)	1.09 (0.86, 1.37)	1.44 (1.11, 1.83)	1.18 (0.89, 1.53)
Severe HFI	1.41 (1.18, 1.66)	1.12 (0.93, 1.34)	1.53 (1.21, 1.91)	1.04 (0.80, 1.34)	1.31 (1.01, 1.66)	1.20 (0.91, 1.55)
	Obesity					
Without HFI	1.00	1.00	1.00	1.00	1.00	1.00
Mild HFI	1.81 (1.35, 2.37)	1.11 (0.81, 1.49)	1.69 (1.17, 2.37)	0.93 (0.62, 1.34)	1.98 (1.21, 3.06)	1.49 (0.88, 2.37)
Moderate HFI	2.02 (1.56, 2.52)	1.21 (0.93, 1.56)	1.74 (1.26, 2.35)	0.99 (0.70, 1.37)	2.44 (1.64, 3.51)	1.67 (1.08, 2.49)
Severe HFI	2.02 (1.58, 2.56)	1.26 (0.96, 1.63)	2.00 (1.44, 2.71)	1.03 (0.71, 1.45)	2.21 (1.50, 3.16)	1.68 (1.11, 2.47)

^1^PR: Prevalence Ratio; ^2^ 95% CI: 95% confidence interval; ^3^ Adjusted for age, sex, and family affluence scale; ^4^ Adjusted for age and family affluence; HFI: household food insecurity; Ref.: reference group.

Although the interaction between sex and HFI was not significant for either overweight (p = 0.51) or obesity (p = 0.08), we also present sex-stratified results in Table 2 and S2 Table. Among boys, we observed a positive association for overweight, although it was not statistically significant after adjustment for age and family affluence. Among girls, we found a statistically significant association between HFI and overweight, which remained after adjustment (PR = 1.26, 95% CI: 1.05, 1.51). By level of HFI, and after adjustment, this association remained statistically significant only for those girls living in mildly food-insecure households (PR = 1.45, 95% CI: 1.08, 1.90).

Similar results were observed for obesity. Among boys, we found no statistically significant associations after adjusting for age and family affluence. However, HFI was associated with obesity among girls (PR = 1.62, 95%CI: 1.16, 2.28). In the adjusted models, the odds of obesity was 67% and 68% higher among girls living in households with moderate HFI (PR = 1.67, 95% CI: 1.08, 2.49) and with severe HFI (PR = 1.68, 95% CI: 1.11, 2.47), respectively, than among girls living in food-secure households.

### Household food insecurity and dietary intake

Table 3 and S3 Table show that children living with HFI were more likely to have a lower intake of fruit than children without HFI.

**Table 3:** Unadjusted and adjusted odds ratios (ORs) and 95% confidence intervals (CIs) for the association of household food insecurity (HFI) with consumption of fruits in children, Madrid, Spain, 2017.

	Overall (n = 4,769)		Boys (n = 2,383)		Girls (n =2,386)	
	Unadjusted	Adjusted^1^	Unadjusted	Adjusted^2^	Unadjusted	Adjusted^2^
	OR (95% CI)	OR (95% CI)	OR (95% CI)	OR (95% CI)	OR (95% CI)	OR (95% CI)
**4-6 times/week (ref. 7 times/week)**						
Without HFI	1.00	1.00	1.00	1.00	1.00	1.00
HFI	1.62 (1.32, 1.99)	1.01 (0.79, 1.29)	1.60 (1.19, 2.14)	1.07 (0.77, 1.49)	1.67 (1.23, 2.27)	1.13 (0.81, 1.58)
**1-3 times/week (ref. 7 times/week)**						
Without HFI	1.00	1.00	1.00	1.00	1.00	1.00
HFI	3.14 (2.52, 3.90)	1.58 (1.23, 2.02)	3.38 (2.49, 4.57)	1.54 (1.08, 2.18)	2.87 (2.15, 3.88)	1.62 (1.17, 2.24)
**Never (ref. 7 times/week)**						
Without HFI	1.00	1.00	1.00	1.00	1.00	1.00
HFI	3.33 (2.39, 4.66)	1.81 (1.25, 2.64)	2.59 (1.58, 4.23)	1.35 (0.77, 2.35)	4.24 (2.67, 6.72)	2.21 (1.27, 3.70)

OR: Odds Ratio; 95% CI: 95% confidence interval; HFI: household food insecurity. ^1^Adjusted by age, sex, and family affluence; ^2^Adjusted by age and family affluence

Table 3 presents sex-stratified results for the association between HFI and fruit consumption (p-interaction = 0.04). Among boys, we observed a statistically significant association only for one category (OR_1–3times/week_ = 1.54, 95% CI: 1.08, 2.18). In contrast, girls living with HFI were 1.62 and 2.21 times more likely to consume fruits 1–3 times/week or never, respectively, compared with those girls without HFI.

For vegetable consumption, as shown in Table 4 and S4 Table, we found similar results. Overall, children with HFI were 3.23 times more likely to never consume vegetables than those without HFI.

**Table 4:** Unadjusted and adjusted odds ratios (ORs) and 95% confidence intervals (CIs) for the association of household food insecurity (HFI) with consumption of vegetables in children, Madrid, Spain, 2017.

	Overall (n = 4,763)		Boys (n = 2,379)		Girls (n = 2,384)	
	Unadjusted	Adjusted^1^	Unadjusted	Adjusted^2^	Unadjusted	Adjusted^2^
	OR (95% CI)	OR (95% CI)	OR (95% CI)	OR (95% CI)	OR (95% CI)	OR (95% CI)
**4-6 times/week (ref. 7 times/week)**						
Without HFI	1.00	1.00	1.00	1.00	1.00	1.00
HFI	1.56 (1.06, 2.29)	1.57 (1.04, 2.38)	1.71 (1.01, 2.91)	1.96 (1.11, 3.52)	1.41 (0.81, 2.46)	1.30 (0.72, 2.35)
**1-3 times/week (ref. 7 times/week)**						
Without HFI	1.00	1.00	1.00	1.00	1.00	1.00
HFI	2.92 (2.02, 4.21)	2.02 (1.36, 2.99)	2.80 (1.61, 4.87)	1.91 (1.10, 3.31)	3.07 (1.81, 5.21)	2.18 (1.24, 3.83)
**Never (ref. 7 times/week)**						
Without HFI	1.00	1.00	1.00	1.00	1.00	1.00
HFI	6.41 (4.19, 9.82)	3.23 (2.03, 5.13)	6.06 (3.32, 11.0)	2.90 (1.51, 5.60)	6.77 (3.69, 12.4)	3.48 (1.81, 6.69)

OR: Odds Ratio; 95% CI: 95% confidence interval; HFI: household food insecurity; Ref.: reference category. ^1^Adjusted by age, sex, and family affluence; ^2^Adjusted by age and family affluence

We also present sex-stratified results, although the interaction term between sex and HFI was not significant (p = 0.15). For both boys and girls, vegetable consumption was lower among children with HFI; however, the direction and pattern of the associations were stronger for girls than for boys (Table 4 and S4 Table).

Table 5 and S5 Table show the overall consumption of SSBs and stratified by sex, although the interaction was not statistically significant (p = 0.93). In the adjusted models, we found a higher probability of having SSBs 7 times a week among children with HFI, compared with those without (OR= 1.67, 95% CI: 1.01, 2.76). Among boys and girls, consumption of SSBs was higher among children with HFI; however, the associations were not statistically significant after adjustment.

**Table 5:** Unadjusted and adjusted odds ratios (ORs) and 95% confidence intervals (CIs) for the association of household food insecurity (HFI) with consumption of sugar-sweetened beverages (SSBs) in children, Madrid, Spain, 2017.

	Overall (n = 4,770)		Boys (n = 2,377)		Girls (n =2,393)	
	Unadjusted	Adjusted^1^	Unadjusted	Adjusted^2^	Unadjusted	Adjusted^2^
	OR (95% CI)	OR (95% CI)	OR (95% CI)	OR (95% CI)	OR (95% CI)	OR (95% CI)
**1-3 times/week (ref. never)**						
Without HFI	1.00	1.00	1.00	1.00	1.00	1.00
HFI	1.82 (1.18, 2.81)	1.32 (0.82, 2.13)	2.13 (1.09, 4.18)	1.25 (0.59, 2.64)	1.61 (0.91, 2.85)	1.34 (0.72, 2.50)
**4-6 times/week (ref. never)**						
Without HFI	1.00	1.00	1.00	1.00	1.00	1.00
HFI	2.95 (1.92, 4.57)	1.53 (0.92, 2.57)	3.60 (1.83, 7.06)	1.48 (0.70, 3.15)	2.50 (1.41, 4.44)	1.50 (0.80, 2.80)
**7 times/week (ref. never)**						
Without HFI	1.00	1.00	1.00	1.00	1.00	1.00
HFI	2.64 (1.67, 4.17)	1.67 (1.01, 2.76)	3.07 (1.51, 6.26)	1.80 (0.79, 4.08)	2.33 (1.26, 4.30)	1.49 (0.76, 2.91)

OR: Odds Ratio; 95% CI: 95% confidence interval; HFI: household food insecurity; Ref.: reference category. ^1^Adjusted by age, sex, and family affluence; ^2^Adjusted by age and family affluence

## Discussion

To the best of our knowledge, this is the first study to assess the association of HFI with childhood obesity and dietary intake by sex/gender in a representative population-based sample of young children in Spain. We found that almost one in five children experienced HFI, underscoring the need to monitor and address HFI in high-income settings. In Spain, a countrywide survey conducted before the COVID-19 pandemic estimated that 13.3% of households experienced HFI [19]. In contrast, a study conducted in Madrid documented a much lower prevalence of HFI (7.7% compared to 18.1% in our study) [26]. However, our prevalence estimate for HFI is similar to that of a study conducted among Spanish adolescents, where 16.2% were classified as food-insecure [56].

We also found that the overall prevalence of obesity was 15.4%; however, this estimate decreased to 13.1% among children without HFI, while it was notably higher among food-insecure households.

Adjusted findings showed a statistically significant association between HFI and overweight but not obesity. Moreover, these associations showed a distinct pattern by sex, with the prevalence of both overweight and obesity being 26% and 62% higher among girls living with HFI than among those who were food-secure. In contrast, HFI was not associated with weight status among boys.

Previous studies have shown inconsistent evidence of the association between HFI and overweight/obesity. A study conducted in the US did not find any association between HFI and anthropometric outcomes [57], whereas other studies conducted outside the US have documented statistically significant associations between HFI and overweight/obesity [26,58]. However, these studies did not present sex-stratified findings.

Interestingly, our findings differed by sex, with associations being more consistent and stronger in girls than in boys. Similarly, a recent systematic review highlighted that when presenting sex-stratified findings, associations between HFI and increased BMI were present in girls but not in boys [24].

As hypothesized, we observed that children experiencing HFI were less likely to consume fruits and vegetables (F&V) daily but more likely to consume SSBs daily. Our findings are consistent with previous findings. In Greece, for example, a study found that children living in food-insecure families had a higher intake of SSBs than children without HFI [33]. A recent US-based review also documented higher SSBs intake among food-insecure youth [59]. Overall, the higher consumption of SBBs in children with HFI is theorized to be driven by families substituting healthier foods (e.g., fruits and vegetables) with less healthy options (e.g., SSBs), which are cheaper.

Few studies have assessed the association of HFI with the consumption of F&V in high-income settings [10,11]. Interestingly, evidence from the US found few differences between children, regardless of HFI status. This may be because families may perceive fruits and vegetables as unaffordable and may substitute them with other foods [60]. For example, based on our findings and those of previous studies, we speculate that food-insecure families may have tried to replace fresh fruits with fruit juices [61,62].

Our sex-stratified analyses also revealed stronger and more consistent associations in girls than in boys for most diet-related outcomes. While no previous study has evaluated the sex-disaggregated association between HFI and dietary outcomes, research has documented that girls have a higher intake of fruits and vegetables than do boys [63]. We hypothesised that sex-related aspects may explain these differences. For example, these healthier eating patterns may reflect the social pressure girls experience from an early age due to gendered norms and expectations.

Since interventions to tackle HFI should be appropriate for all children and adolescents regardless of sex, more epidemiologists are calling for routinely providing sex-disaggregated on associations and to evaluate sex specific aspects [64,65]. Unveiling these differences should lead to further investigation into their causes in future research. For example, researchers have considered that the differential responses to stress between males and females could lead to lower stress/anxiety due to food insecurity, and consequently less negative effects on health outcomes [35]. While participants were only 3–12 years old, and thus too young to be very aware, gendered roles within households could influence children’s physical health and mental well-being. In addition, it is important to further explore whether caregivers’ feeding practices vary depending on the child’s sex.

Our study had some limitations that should be considered when interpreting the findings. First, we present cross-sectional evidence; thus, we cannot infer temporality or causality. Second, food insecurity was measured at the household-level and may not capture the food security status of each household member. Notably, previous research has shown differences in intra-household food allocation based on sex and age [38]. For example, younger children are often protected by adults over older children [10]. Additionally, mothers often prioritize feeding their children before themselves to protect them from the effects of household food insecurity [39]. While this might bias the number of individuals experiencing food insecurity within each household, it still provides an accurate estimate of the number of households with at least one member experiencing food insecurity. Third, measures of dietary intake were self-reported by parents, which might have introduced recall and social desirability biases. Finally, we did not consider mental health outcomes, which have been previously associated with HFI in high-income countries [66,67].

Despite these limitations, this study relied on a validated and widely used measure of HFI, which allowed us to differentiate across four levels of household food security. Anthropometric data were objectively assessed by trained staff according to standard protocols. In addition, we had a representative sample of children aged 3–12 years in Madrid, highlighting the relevance of these analyses and results.

Our study has several public health implications. By improving children’s access to healthy and nutritious food, it may be possible to simultaneously reduce household food insecurity and the risk of obesity, in both the short- and long-term. Current household- or school-level policies, such as SNAP or universal school meals, have proven successful and equitable in reducing food insecurity [3,68]. Our study highlights the need to monitor HFI as a key step to inform public health interventions in Spain, where no explicit policy addresses food insecurity.

## Conclusions

Household food insecurity was experienced by 18.1% of children aged 3–12 years living in Madrid, Spain. Children living in food-insecure households had a higher prevalence of overweight but not obesity, less frequent consumption of fruits and vegetables and higher odds of daily intake of sugar-sweetened beverages. Sex-stratified analyses showed stronger and more consistent associations in girls than in boys. Our findings support the need for routinely monitoring household food insecurity and the development of policies ensuring stable access to healthy and nutritious foods among

## Supporting information

S1 Fig Flowchart of the study participants. (PDF)

S2 Fig Classification of household food insecurity into levels (adapted from Coates et al., 2007). (PDF)

S1 Table Sample characteristics by household food insecurity (n = 5,144). (PDF)

S2 Table Prevalence ratios (PR) of overweight and obesity by household food insecurity among boys and girls (n = 4,241). (PDF)

S3 Table Odds ratios for the association between food insecurity and frequency of fruit consumption among children. (PDF)

S4 Table Odds ratios for the association between food insecurity and frequency of vegetable consumption among children. (PDF)

S5 Table Odds ratios for the association between food insecurity and frequency of sugar-sweetened beverage (SSB) consumption among children. (PDF)

S1 File STROBE statement. (PDF)
