# Supplementary material for: Household food insecurity, dietary intake, and weight status among children in Madrid, Spain: A cross-sectional study
Source: PLoS One. 2026 Sep 30;21(9):e0358967. doi: 10.1371/journal.pone.0358967 (PMC13626367; doi:10.1371/journal.pone.0358967)

S2 Figure. Classification of household food insecurity into levels (adapted from Coates et al., 2007)

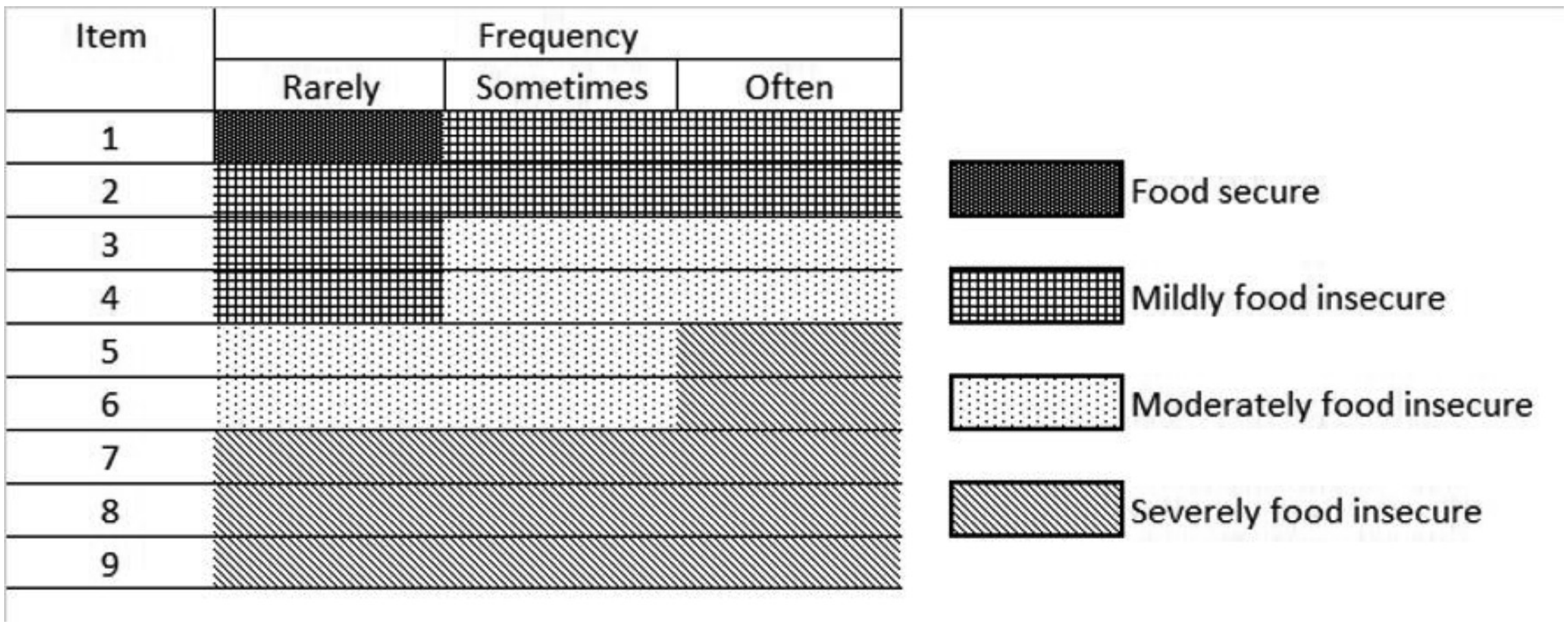

Supplement: Supplementary file 2 [file pone.0358967.s002.pdf]
