# Supplementary material for: Household food insecurity, dietary intake, and weight status among children in Madrid, Spain: A cross-sectional study
Source: PLoS One. 2026 Sep 30;21(9):e0358967. doi: 10.1371/journal.pone.0358967 (PMC13626367; doi:10.1371/journal.pone.0358967)
Supplement: Supplementary file 3 [file pone.0358967.s003.pdf]

Table S1. Sample characteristics by household food insecurity (n = 5,144)

	Total (N; %) (5144; 100.0)		Food-secure (N; %) (4168; 81.9)		Food-insecure (N; %) (976; 18.1)	
**Age, (mean, SD[1])**	7.28 (2.83)		7.18 (2.82)		7.70 (2.88)	
**Sex, % (95% CI[2])**						
Girls	48.6	(47, 50)	48.5	(46, 51)	49	(44, 54)
**Family Affluence Scale, % (95% CI[2])**						
Low	40.7	(39, 42)	30.5	(28, 33)	88.4	(85, 91)
Medium	34.1	(33, 36)	39.4	(37, 42)	9.1	(7, 12)
High	25.3	(24, 27)	30.1	(28, 32)	2.5	(1, 5)
**Body weight, % (95% CI[2]**						
Overweight	25.4	(24, 27)	24.4	(23, 26)	29.9	(25, 35)
Obesity	15.4	(14, 17)	13.1	(12, 15)	25.6	(22, 30)
**Fruit consumption, % (95% CI[2])**						
Every day	32.6	(31, 34)	35.4	(33, 38)	19.5	(16, 24)
**Vegetables consumption, % (95% CI[2])**						
Every day	8.2	(7, 9)	9.1	(8, 11)	3.9	(3, 6)
**SSB[3] consumption, % (95% CI[2])**						
Never	6.3	(6, 7)	7.1	(6, 8)	2.7	(2, 4)
