# Supplementary material for: Household food insecurity, dietary intake, and weight status among children in Madrid, Spain: A cross-sectional study
Source: PLoS One. 2026 Sep 30;21(9):e0358967. doi: 10.1371/journal.pone.0358967 (PMC13626367; doi:10.1371/journal.pone.0358967)
Supplement: Supplementary file 4 [file pone.0358967.s004.pdf]

S2 Table**.** Prevalence ratios (PR) of overweight and obesity by household food insecurity among boys and girls (n = 4,241)

		**Overweight, PR[1] (95% IC)[2]**		
		Model 1[3]	Model 2[4]	Model 3[5]
Overall	*No HFI*	Ref.	Ref.	Ref.
	HFI	**1.50 (1.35, 1.66)**	**1.47 (1.33, 1.63)**	**1.17 (1.04, 1.31)**
		**Obesity, PR[1] (95% IC)[2]**		
	No HFI	Ref.	Ref.	Ref.
	HFI	**1.97 (1.63, 2.39)**	**1.90 (1.57, 2.31)**	1.20 (0.97,1.48)
		**Overweight , PR[1] (95% IC)[2]**		
		Model 1[3]	Model 2[4]	Model 3[5]
Boys	*No HFI*	Ref.	Ref.	Ref.
	HFI	**1.48 (1.30, 1.69)**	**1.43 (1.24, 1.64)**	1.08 (0.94, 1.25)
		**Obesity , PR[1] (95% IC)[2]**		
	No HFI	Ref.	Ref.	Ref.
	HFI	**1.76 (1.37, 2.24)**	**1.67 (1.30, 2.13)**	0.99 (0.76, 1.28)
		**Overweight, PR[1] (95% IC)[2]**		
		Model 1[3]	Model 2[4]	Model 3[5]
Girls	*No HFI*	Ref.	Ref.	Ref.
	HFI	**1.53 (1.30, 1.79)**	**1.52 (1.30, 1.78)**	**1.26 (1.05, 1.51)**
		**Obesity, PR[1] (95% IC)[2]**		
	No HFI	Ref.	Ref.	Ref.
	HFI	**2.37 (1.74, 3.23)**	**2.35 (1.73, 3.19)**	**1.62 (1.16, 2.28)**
