# Supplementary material for: Household food insecurity, dietary intake, and weight status among children in Madrid, Spain: A cross-sectional study
Source: PLoS One. 2026 Sep 30;21(9):e0358967. doi: 10.1371/journal.pone.0358967 (PMC13626367; doi:10.1371/journal.pone.0358967)
Supplement: Supplementary file 6 [file pone.0358967.s006.pdf]

S4 Table. Odds ratios for the association between food insecurity and frequency of vegetable consumption among children.

| | Always vs | 4-6 times per week | | 1-3 times a week | | Never | |
|---|---|---|---|---|---|---|---|
| | | Unadjusted OR[1] (95% CI[2]) | Adjusted[3] OR (95% CI) | Unadjusted OR (95% CI) | Adjusted[4] OR (95% CI) | Unadjusted OR (95% CI) | Adjusted[4] OR (95% CI) |
| Overall | No HFI | REF. | REF. | REF. | REF. | REF. | REF. |
| | HFI Midly | 1.48 (0.72, 3.08) | 1.52 (0.72, 3.19) | 2.66 (1.33, 5.32) | 1.90 (0.94, 3.87) | 4.45 (1.95, 10.2) | 2.32 (0.99, 5.41) |
| | HFI Moderately | 1.79 (0.83, 3.86) | 1.84 (0.83, 4.06) | 4.35 (2.09, 9.05) | 2.95 (1.39, 6.26) | 8.09 (3.56, 18.4) | 3.76 (1.62, 8.77) |
| | HFI Severe | 1.27 (0.69, 2.35) | 1.33 (0.70, 2.52) | 2.01 (1.12, 3.59) | 1.36 (0.74, 2.51) | 5.51 (2.82, 10.8) | 2.54 (1.25, 5.14) |
| | Always vs | 4-6 times per week | | 1-3 times a week | | Never | |
| | | Unadjusted OR[1] (95% CI[2]) | Adjusted[3] OR (95% CI) | Unadjusted OR (95% CI) | Adjusted[4] OR (95% CI) | Unadjusted OR (95% CI) | Adjusted[4] OR (95% CI) |
| Boys | No HFI | REF. | REF. | REF. | REF. | REF. | REF. |
| | HFI Midly | 1.92 (0.59, 6.22) | 2.34 (0.70, 7.81) | 3.87 (1.26,11.9) | 2.72 (0.86, 8.61) | 5.25 (1.39, 19.8) | 2.30 (0.59, 8.95) |
| | HFI Moderately | 2.40 (0.79, 7.28) | 2.90 (0.92, 9.11) | 4.99 (1.71, 14.5) | 3.37 (1.12, 10.1) | 4.98 (1.39, 17.9) | 2.08 (0.56, 7.75) |
| | HFI Severe | 1.12 (0.51, 2.49) | 1.38 (0.59, 3.23) | 1.38 (0.65, 2.95) | 0.95 (0.42, 2.12) | 4.07 (1.64, 10.1) | 1.69 (0.64, 4.42) |
| | Always vs | 4-6 times per week | | 1-3 times a week | | Never | |
| | | Unadjusted OR[1] (95% CI[2]) | Adjusted[3] OR (95% CI) | Unadjusted OR (95% CI) | Adjusted[4] OR (95% CI) | Unadjusted OR (95% CI) | Adjusted[4] OR (95% CI) |
| Girls | No HFI | REF. | REF. | REF. | REF. | REF. | REF. |
| | HFI Midly | 1.23 (0.48, 3.13) | 1.16 (0.45, 3.00) | 1.93 (0.79, 4.69) | 1.45 (0.59, 3.58) | 3.95 (1.37, 11.4) | 2.24 (0.76, 6.64) |
| | HFI Moderately | 1.26 (0.43, 3.71) | 1.19 (0.39, 3.60) | 3.75 (1.37, 10.3) | 2.64 (0.94, 7.42) | 10.6 (3.54, 31.5) | 5.23 (1.69, 16.2) |
| | HFI Severe | 1.51 (0.57, 3.97) | 1.42 (0.52, 3.86) | 3.04 (1.21, 7.62) | 2.07 (0.80, 5.37) | 7.73 (2.79, 21.5) | 3.83 (1.32, 11.1) |
