# Supplementary material for: Household food insecurity, dietary intake, and weight status among children in Madrid, Spain: A cross-sectional study
Source: PLoS One. 2026 Sep 30;21(9):e0358967. doi: 10.1371/journal.pone.0358967 (PMC13626367; doi:10.1371/journal.pone.0358967)
Supplement: Supplementary file 7 [file pone.0358967.s007.pdf]

S5 Table. Odds ratios for the association between food insecurity and frequency of sugar-sweetened beverage (SSB) consumption among children

	Never vs	1-3 times a week		4-6 times per week		Always	
		Unadjusted OR[1] (95% CI[2])	Adjusted[3] OR (95% CI)	Unadjusted OR (95% CI)	Adjusted[4] OR (95% CI)	Unadjusted OR (95% CI)	Adjusted[4] OR (95% CI)
Overall	No HFI	REF.	REF.	REF.	REF.	REF.	REF.
	HFI Midly	2.22 (0.93, 5.27)	1.69 (0.70, 4.11)	3.38 (1.42, 8.07)	1.85 (0.76, 4.51)	2.56 (1.02, 6.43)	1.78 (0.69, 4.57)
	HFI Moderately	2.48 (1.12, 5.50)	1.80 (0.79, 4.10)	3.02 (1.35, 6.75)	1.47 (0.64, 3.39)	4.22 (1.85, 9.59)	2.66 (1.13, 6.27)
	HFI Severe	1.60 (0.77, 3.31)	1.11 (0.51, 2.38)	2.91 (1.41, 6.03)	1.36 (0.63, 2.93)	2.5 (1.19, 5.52)	1.60 (0.72, 3.60)

	Never vs	1-3 times a week		4-6 times per week		Always	
		Unadjusted OR[1] (95% CI[2])	Adjusted[3] OR (95% CI)	Unadjusted OR (95% CI)	Adjusted[4] OR (95% CI)	Unadjusted OR (95% CI)	Adjusted[4] OR (95% CI)
Boys	No HFI	REF.	REF.	REF.	REF.	REF.	REF.
	HFI Midly	1.06 (0.41, 2.75)	0.62 (0.22, 1.72)	2.04 (0.79, 5.25)	0.80 (0.29, 2.21)	1.05 (0.36, 3.06)	0.64 (0.21, 1.98)
	HFI Moderately	8.53 (1.11, 65.3)	5.01 (0.63, 39.8)	9.64 (1.25, 74.2)	3.65 (0.46, 29.2)	16.0 (2.05, 124)	9.39 (1.16, 76.1)
	HFI Severe	1.62 (0.55, 4.75)	0.91 (0.29, 2.87)	2.48 (0.84, 7.31)	0.92 (0.29, 2.90)	2.46 (0.80, 7.61)	1.44 (0.43, 4.79)

	Never vs	1-3 times a week		4-6 times per week		Always	
		Unadjusted OR[1] (95% CI[2])	Adjusted[3] OR (95% CI)	Unadjusted OR (95% CI)	Adjusted[4] OR (95% CI)	Unadjusted OR (95% CI)	Adjusted[4] OR (95% CI)
Girls	No HFI	REF.	REF.	REF.	REF.	REF.	REF.
	HFI Midly	11.8 (0.93, 150)	10.3 (0.80, 131)	13.8 (1.07, 177)	9.26 (0.72, 120)	15.8 (1.20, 207)	11.5 (0.87, 153)
	HFI Moderately	1.45 (0.60, 3.51)	1.17 (0.47, 2.96)	1.91 (0.78, 4.69)	1.11 (0.43, 2.82)	2.09 (0.80, 5.45)	1.34 (0.49, 3.65)
	HFI Severe	1.58 (0.59, 4.26)	1.22 (0.43, 3.42)	3.37 (1.26, 9.01)	1.81 (0.65, 5.04)	2.69 (0.94, 7.68)	1.63 (0.55, 4.89)
