# Supplementary material for: Household food insecurity, dietary intake, and weight status among children in Madrid, Spain: A cross-sectional study
Source: PLoS One. 2026 Sep 30;21(9):e0358967. doi: 10.1371/journal.pone.0358967 (PMC13626367; doi:10.1371/journal.pone.0358967)
Supplement: Supplementary file 8 [file pone.0358967.s008.pdf]

STROBE Statement—Checklist of items that should be included in reports of ***cross-sectional studies***

	Item No	Recommendation	Location in manuscript where items are reported
**Title and abstract**	1	(*a*) Indicate the study's design with a commonly used term in the title or the abstract	Title, page 1; Abstract, page 2
		(*b*) Provide in the abstract an informative and balanced summary of what was done and what was found	Abstract, pages 2–3
**Introduction**			
Background/rationale	2	Explain the scientific background and rationale for the investigation being reported	Lines 1–50
Objectives	3	State specific objectives, including any prespecified hypotheses	Lines 51–58
**Methods**			
Study design	4	Present key elements of study design early in the paper	Line 59-63
Setting	5	Describe the setting, locations, and relevant dates, including periods of recruitment, exposure, follow-up, and data collection	Line 61-65
Participants	6	(*a*) Give the eligibility criteria, and the sources and methods of selection of participants	Line 67-83
Variables	7	Clearly define all outcomes, exposures, predictors, potential confounders, and effect modifiers. Give diagnostic criteria, if applicable	Lines 91- 157
Data sources/ measurement	8*	For each variable of interest, give sources of data and details of methods of assessment (measurement). Describe comparability of assessment methods if there is more than one group	Lines 91- 157
Bias	9	Describe any efforts to address potential sources of bias	Line 92-95; lines 109-114; lines 162-163
Study size	10	Explain how the study size was arrived at	Lines 67-83 and Figure S1
Quantitative variables	11	Explain how quantitative variables were handled in the analyses. If applicable, describe which groupings were chosen and why	Line 115 -123, 142, 150-157
Statistical methods	12	(*a*) Describe all statistical methods, including those used to control for confounding	Lines 158-181
		(*b*) Describe any methods used to examine subgroups and interactions	Lines 169- 177
		(*c*) Explain how missing data were addressed	Lines 74-83 Figure S1
		(*d*) If applicable, describe analytical methods taking account of sampling strategy	No applicable
		(*e*) Describe any sensitivity analyses	Lines 175-177
**Results**			
Participants	13*	(a) Report numbers of individuals at each stage of study—eg numbers potentially eligible, examined for eligibility, confirmed eligible, included in the study, completing follow-up, and analysed	Line 67-83

		(b) Give reasons for non-participation at each stage	No information
		(c) Consider use of a flow diagram	Figure S1
Descriptive data	14*	(a) Give characteristics of study participants (eg demographic, clinical, social) and information on exposures and potential confounders	Lines 182 -199
		(b) Indicate number of participants with missing data for each variable of interest	Figure S1
Outcome data	15*	Report numbers of outcome events or summary measures	Lines 194 -199
Main results	16	(*a*) Give unadjusted estimates and, if applicable, confounder-adjusted estimates and their precision (eg, 95% confidence interval). Make clear which confounders were adjusted for and why they were included	Lines 200-267; Tables 2,3,4,5
		(*b*) Report category boundaries when continuous variables were categorized	No applicable
		(*c*) If relevant, consider translating estimates of relative risk into absolute risk for a meaningful time period	No applicable
Other analyses	17	Report other analyses done—eg analyses of subgroups and interactions, and sensitivity analyses	Supplemental material
**Discussion**			
Key results	18	Summarise key results with reference to study objectives	Lines 276-327
Limitations	19	Discuss limitations of the study, taking into account sources of potential bias or imprecision. Discuss both direction and magnitude of any potential bias	Lines 340- 354
Interpretation	20	Give a cautious overall interpretation of results considering objectives, limitations, multiplicity of analyses, results from similar studies, and other relevant evidence	Lines 276-327
Generalisability	21	Discuss the generalisability (external validity) of the study results	Lines 355-360
**Other information**			
Funding	22	Give the source of funding and the role of the funders for the present study and, if applicable, for the original study on which the present article is based	Lines 61 – 64; Lines 388-389

*Give information separately for exposed and unexposed groups.

Note: An Explanation and Elaboration article discusses each checklist item and gives methodological background and published examples of transparent reporting. The STROBE checklist is best used in conjunction with this article (freely available on the Web sites of PLoS Medicine at http://www.plosmedicine.org/, Annals of Internal Medicine at http://www.annals.org/, and Epidemiology at http://www.epidem.com/). Information on the STROBE Initiative is available at www.strobe-statement.org.
